# Targeting the autosomal *Ceratitis capitata transformer* gene using Cas9 or dCas9 to masculinize XX individuals without inducing mutations

**DOI:** 10.1186/s12863-020-00941-4

**Published:** 2020-12-18

**Authors:** Pasquale Primo, Angela Meccariello, Maria Grazia Inghilterra, Andrea Gravina, Giuseppe Del Corsano, Gennaro Volpe, Germano Sollazzo, Serena Aceto, Mark D. Robinson, Marco Salvemini, Giuseppe Saccone

**Affiliations:** 1 Department of Biology, University of Naples Federico II, 80126 Naples, Italy; 2 Department of Molecular Life Sciences and SIB Swiss Institute of Bioinformatics, University of Zurich, Winterthurerstrasse 190, CH-8057 Zurich, Switzerland

**Keywords:** iCRISPR, Sex determination, *Ceratitis capitata*, Epigenetics, Autoregulation, *Transformer*

## Abstract

**Background:**

Females of the Mediterranean fruit fly *Ceratitis capitata* (Medfly) are major agricultural pests, as they lay eggs into the fruit crops of hundreds of plant species. In Medfly, female sex determination is based on the activation of *Cctransformer* (*Cctra*). A maternal contribution of *Cctra* is required to activate *Cctra* itself in the XX embryos and to start and epigenetically maintain a *Cctra* positive feedback loop, by female-specific alternative splicing, leading to female development. In XY embryos, the male determining *Maleness-on-the-Y* gene (*MoY*) blocks this activation and *Cctra* produces male-specific transcripts encoding truncated CcTRA isoforms and male differentiation occurs.

**Results:**

With the aim of inducing frameshift mutations in the first coding exon to disrupt both female-specific and shorter male-specific CcTRA open reading frames (ORF), we injected Cas9 ribonucleoproteins (Cas9 and single guide RNA, sgRNA) in embryos. As this approach leads to mostly monoallelic mutations, masculinization was expected only in G_1_ XX individuals carrying biallelic mutations, following crosses of G_0_ injected individuals. Surprisingly, these injections into XX-only embryos led to G_0_ adults that included not only XX females but also 50% of reverted fertile XX males. The G_0_ XX males expressed male-specific *Cctra* transcripts, suggesting full masculinization. Interestingly, out of six G_0_ XX males, four displayed the *Cctra* wild type sequence. This finding suggests that masculinization by Cas9-sgRNA injections was independent from its mutagenic activity. In line with this observation, embryonic targeting of *Cctra* in XX embryos by a dead Cas9 (enzymatically inactive, dCas9) also favoured a male-specific splicing of *Cctra*, in both embryos and adults.

**Conclusions:**

Our data suggest that the establishment of *Cctra* female-specific autoregulation during the early embryogenesis has been repressed in XX embryos by the transient binding of the Cas9-sgRNA on the first exon of the *Cctra* gene. This hypothesis is supported by the observation that the shift of *Cctra* splicing from female to male mode is induced also by dCas9. Collectively, the present findings corroborate the idea that a transient embryonic inactivation of *Cctra* is sufficient for male sex determination.

**Supplementary Information:**

The online version contains supplementary material available at 10.1186/s12863-020-00941-4.

## Background

In the last few decades, the Mediterranean fruit fly *Ceratitis capitata* (Tephritidae, Medfly) has become a major invasive agricultural pest worldwide, following its spread from Africa and its globalization [1]. For the local suppression of this invasive species, alternatives to the use of pesticides are genetic control strategies. One of them is the Sterile Insect Technique (SIT), which has been applied successfully over the last six decades in various countries [2].

**Fig. 1:**
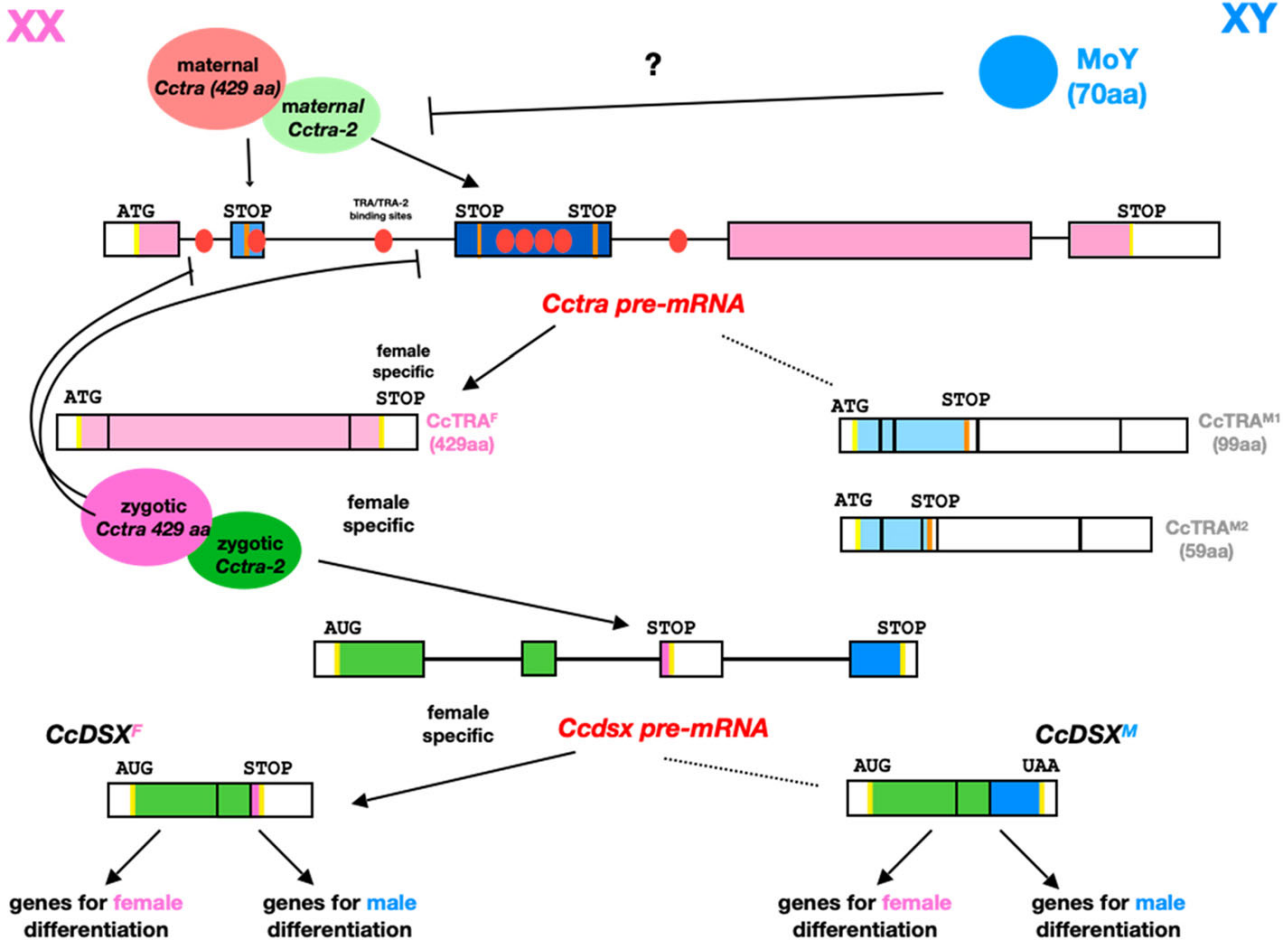
Genetic pathway of sex determination in *Ceratitis capitata*. *Cctra* and *Ccdoublesex* (*Ccdsx*) pre-mRNAs exon-intron structures and sex-specific transcripts are shown. Female-specific and male-specific *Cctra* exons are indicated as pink and dark blue boxes, respectively. *Cctra* female-specific transcript on the left contains a 429 aa long ORF. *Cctra* male-specific exons introduce premature stop codons in male-specific longer transcripts (orange vertical bars). CcTRA M1 and M2 male-specific isoforms contain truncated CcTRA ORFs represented by azul regions. In XX embryos, maternal CcTRA (orange circle) and CcTRA-2 (green circle) proteins promote female-specific splicing of newly transcribed *Cctra* pre-mRNA, suppressing male-specific splicing by binding to TRA/TRA-2 *cis* regulatory elements (red spots). Female-specific *Cctra* mRNA encodes zygotic CcTRA (violet circle) that maintains (together with zygotic CcTRA-2; dark green circle) the *Cctra* autoregulation induced by the maternal contributions by a feedback loop. Both CcTRA and CcTRA-2 proteins promote also female-specific splicing of the downstream *Ccdsx* pre-mRNA, producing mRNAs that include a female-specific exon (pink) and encode CcDSXF isoform inducing female sexual differentiation [6]. In XY embryos the Y-linked *Maleness-on-the-Y* gene (*MoY*) induces male-specific *Cctra* splicing and, hence, the collapse of the positive feedback loop [7]. By default, male-specific splicing of *Ccdsx* leads to male-specific splicing and CcDSM isoform inducing male sexual differentiation [6, 8]

The prerequisites of SIT include a method to mass rear the target species in a cost-effective way and a method to sterilize them with a low impact on their fitness once released. As the released sterile females contribute to the fruit crop mechanical damage with the ovipositor and consequent infections, and the sterile males tend to mate with the released females rather than with the wild ones, it is highly preferable to develop a method of sexing and only release sterile males [3]. A number of strategies have been developed, including transgenic approaches for sexing, which allow the mass rearing of the two sexes, and sorting the males at the expanded last generation before the release. These strains can be based on the expression of a conditional female-lethal dominant gene [4] or on the transformation of genotypic female individuals into males by manipulating a gene involved in female sex determination [5]. Molecular genetics studies on Medfly sex determination have been useful for this aim, uncovering a cascade of regulatory genes widely conserved in the Tephritidae family (Fig. 1) [6–10]. This taxon includes many other invasive agricultural pests, such as species of the *Bactrocera* and *Anastrepha* genera [11, 12]. This fundamental knowledge is not only interesting and valuable per se [13], but also useful to develop novel sexing strategies necessary to improve the applicability of SIT. Evolutionary conservation of homologous genes and the use of transgenesis and/or CRISPR/Cas9 potentially will enable the realization of additional versatile sexing methods that can be applied in different species [8, 9, 14–16].

The sex determination of Medfly is based, as in *Drosophila melanogaster*, on sex-specific alternative splicing of key regulatory genes, including *transformer* (*tra*), *transformer-2* (*tra-2*) and *doublesex* (*dsx*) orthologues (Fig. 1) [6, 7, 17–20]. *Cctra* is a sex determining genetic switch, which is set to ON in XX embryos and to OFF in XY embryos during a narrow temporal window at 5–6 h from oviposition [8]. In contrast to *Drosophila*, in XX Medfly embryos, *Cctra* and the auxiliary *Cctransformer-2* (*Cctra-2*) maternal mRNAs are also required to establish a stable activation of *Cctra* by female-specific splicing which relies on a positive feedback loop [6, 19]. The female-specific *Cctra* mRNA encodes a full-length 429 aa protein arising from the translation of an evolutionarily conserved ORF contained in the first, fourth and fifth exons (Fig. 1). In males, two longer alternative *Cctra* RNA isoforms, containing all five exons, encode for two truncated CcTRA proteins called CcTRA M1 (59 aa) and CcTRA M2 (99 aa) (Fig. 1). Embryonic transient RNA interference targeting mRNAs of either genes led to XX males, which are fertile even in the absence of the Y chromosome [6, 19]. As in *Drosophila*, *Cctra* and *Cctra-2* are required for the female-specific splicing of *Ccdoublesex* (*Ccdsx*). The observation that some XX individuals are transformed in gynandromorphs (showing male-specific antennae and ovipositor or no antennae and male gonads) suggests that sex determination is cell-autonomous, as in *Drosophila* [6]. A TRA/TRA-2 binding element (13 nt long) is present in multiple copies in the *Ccdsx* female-specific exon, permitting a positive regulation by CcTRA/CcTRA-2, which leads to the use of this exon in *Ccdsx* transcripts of XX individuals, similarly to *Drosophila* [7, 18]. In contrast to *Drosophila*, multiple copies of this splicing regulatory element are also present in *Cctra* locus, within and in proximity to the male-specific exons. In this other novel case, these cis elements mediate, by CcTRA/CcTRA-2 binding, exon skipping in XX individuals leading to CcTRA-encoding female-specific mRNAs [6]. In XY embryos, *Maleness-on-the-Y* (*MoY*) encodes a novel short protein, MOY, of still unknown biochemical function, that leads, either directly or indirectly, to male-specific splicing of *Cctra* and exons inclusion*,* at 5–6 h from oviposition [8]. The presence of male-specific exons introducing stop codons in the 429 aa long *Cctra* ORF leads to two major RNAs encoding truncated polypeptides (respectively 59 and 99 aa long) and hence considered to be non-functional [6].

We planned to experimentally confirm this deduction, by Cas9-induced mutations in the *Cctra*, which would impact both the male- and female-specific ORFs. Furthermore, the availability of an efficient single RNA to induce mutations in a female-determining gene would open the future possibility to develop a gene drive strategy aimed at manipulating the sex ratio and hence the reproduction rate of this harmful species [4]. CRISPR/Cas9 has been used in the Medfly genome to target autosomal genes having two copies for each cell [16, 21, 22]. After targeting the *white eye* Medfly gene by injecting into early embryos in vitro pre-assembled and solubilized Cas9 ribonucleoprotein complexes (RNPs), containing sgRNA, adults showed partial mutant phenotypes caused by somatic mosaicism [16]. The most extreme mutant phenotype consisted of a fly mosaic with one of the two eyes fully colorless. While biallelic mutations were observed only in somatic clones of the fly, the germ line transmission rate was very high, reaching 100% in one case. On the contrary, targeting a Medfly single copy gene, as the Y-linked *MoY,* in XY individuals by Cas9 ribonucleoproteins injections, 70% of mutant G_0_ individuals showed intersexual phenotype and 30% were transformed into XY mutant G_0_ females [8].

We reasoned that introducing loss-of-function frameshift mutations in the first *Cctra* exon with CRISPR/Cas9 would lead to mutant alleles coding for truncated CcTRA proteins. The expected truncations would affect not only the 429 aa long female-specific ORF, but also the carboxy-terminal ends of two male-specific 59 and 99 aa ORFs. A masculinization of XX individuals by permanent loss-of-function mutations of *Cctra* altering also the male-specific CcTRA polypeptides would support the previous suggestion that these products are indeed non-functional.

**Fig. 2:**
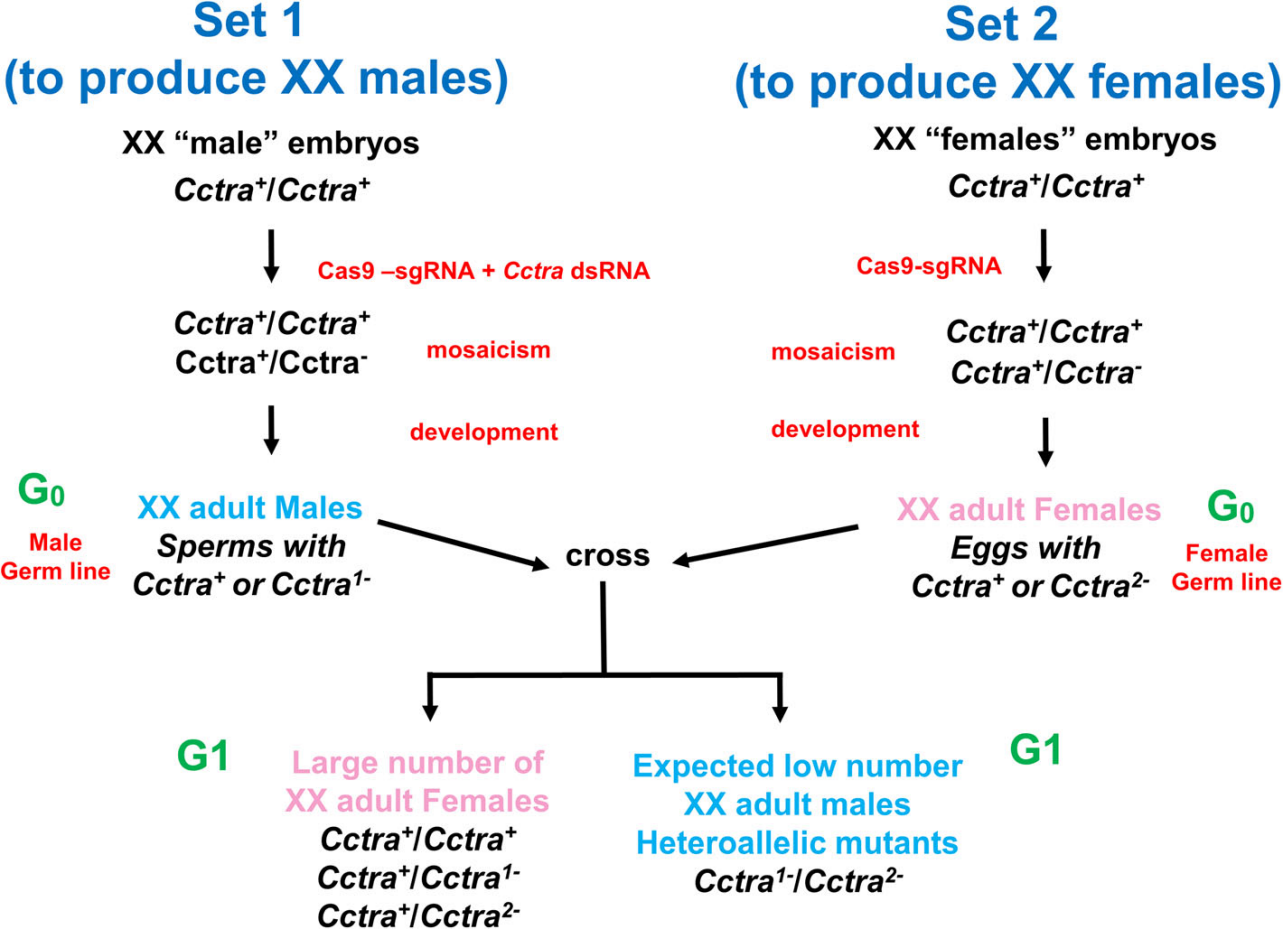
Experimental design to produce G_1_ XX males bearing heteroallelic mutation of *Cctra*. Two sets of parallel Cas9-RNP injections to target *Cctra* into XX embryos can lead to both G_0_ XX males and XX females. The G_0_ adults from the two sets can bear some *Cctra* monoallelic independent mutations (mosaicism) both in the soma and in the germ line. In set n. 1, the injection mix contains also *Cctra*-dsRNA to induce full masculinization of XX embryos (Pane et al., 2002). Hence, the G_0_ XX males from set n. 1 can be crossed with G_0_ XX females from set n. 2, to produce a G_1_ XX progeny, expected to be composed mostly of XX females (carrying either none or monoallelic mutations) and of few G_1_ XX males, carrying heteroallelic mutations of *Cctra* (*Cctra*^*1−*^/*Cctra*^*2−*^) inherited from the respective parents

Since in *Drosophila tra* and *tra-2* mutant alleles are recessive, we reasoned that also in the Medfly the presence in the same cell and in its clonal descendants of only monoallelic indel (insertion/deletion) mutations in *Cctra* (+/− heterozygous state) would be insufficient to masculinize XX cells. Thus, in the somatic mutant clones of these XX individuals, the CcTRA protein expressed from the wild-type allele would lead to female-specific splicing of *Cctra* pre-mRNAs from both wild-type and mutant alleles, leading to female sexual phenotype. Hence, only biallelic loss-of-function *Cctra* mutations (Fig. 2; *Cctra*^*1−*^/*Cctra*^*2−*^) in the same XX cell and its cellular descendants would lead to a male-specific *Cctra* and the downstream *Ccdsx* RNAs, causing a partial (mosaicism) or full (very early and high bi-allelic mutagenesis) masculinization of XX adults.

## Results

### Cas9-RNP injections targeting *Cctra* lead to fully masculinized XX flies in the G_0_ progeny

With the aim of inducing indels leading to frameshift mutations in *Cctra*, we used Cas9 RNPs injections as a delivery method. In particular, a CRISPR/Cas9 *Cctra* target site was chosen within the coding region of exon 1 on the antisense strand to design a single guide RNA, named sgtraEx1 (Additional file 1: Fig. S1A). The targeted 20 bp long sequence is about 20 bp upstream of the first donor splicing site and upstream to the female-specific long open reading frame encoding the CcTRA protein (Additional file 1: Fig. S1B). As described above, previous literature data showed that Cas9 RNPs applied to Medfly embryos led to a relatively low somatic biallelic mutation rate overall but a high rate in the germ line [16]. Based on this study, we speculated to observe masculinization of XX *Cctra* mutant individuals only at G_1_ after crossing G_0_ XY males and G_0_ XX females developed from Cas9-injected. However, the discrimination of rare *Cctra*^*1−*^/*Cctra*^*2−*^ heteroallelic mutant XX males among the 50% of the G_1_ progeny being XY males would have been tedious and challenging. We simplified their identification by planning to detect them among a G_1_ female-only XX progeny, obtained crossing XX males and females obtained from RNP-injected embryos (Fig. 2). Among these G_1_ XX females, *Cctra*^*-1−*^/*Cctra*^*2−*^ XX males would be easily detected even if very few, with the respect of the majority of XX females carrying either none or only one *Cctra* mutant allele (Fig. 2).

To produce a larger number of XX males (Fig. 2, set 1), from Cas9-injected XX embryos, a mix of Cas9-sgtraEx1 RNP and *Cctra* dsRNA, to efficiently masculinize XX, was injected into 370 XX embryos (Table 1, set n. 1). The aim was to obtain XX fertile males potentially carrying Cas9-induced *Cctra* monoallelic mutations also in the germ line. However, no pupae and adult flies emerged from this set of injections. When Cas9-sgtraEx1 RNP alone targeting *Cctra* were delivered into 400 XX embryos, few adults developed (3%; 12/400) (Table 1, set n. 2). It is likely that the co-presence of dsRNA+Cas9/sgRNA molecules could have a combined higher lethal effect for unclear reasons. Injections of dsRNA-*Cctra* alone resulted in 78 adults out of 200 XX embryos injected, with 73 XX being masculinized individuals and 5 being intersexes (Table 1, set n. 3). A similar survival rate was obtained when we injected sgtraEx1 RNA molecules but no effect (1 μg/μL), at a 5 times higher concentration being more susceptible to RNA degradation (70 females out of 160 injected embryos; Table 1, set n. 4).

**Table 1:** XX-only embryonic injection sets

Injection set	Cas9 delivery / []	sgRNA name / []	dsRNA name / []	XX embryos	XX Larvae	XX Females	XX Males	XX intersexes	***Cctra*** male-specific in XX
1	Cas9 Protein / 1,8 μg/μL	sgtraEx1 / 200 ng/μL	dsRNA-Cctra / 0.5 μg/μL	370	48	0	0	0	–
2	Cas9 Protein / 1,8 μg/μL	sgtraEx1 / 200 ng/μL	–	400	60	6	6	0	yes in XX males
3	–	–	dsRNA-Cctra / 0.5 μg/μL	200	83	0	73	5	–
4	–	sgtraEx1 / 1 μg/μL	–	160	75	70	0	0	no
5	Plasmid-dCas9 / 1 μg/μL	sgtraEx1 / 1 μg/μL	–	290	37	7	0	3	yes
6	Cas9 Protein / 1,8 μg/μL	sgtraEx1 / 200 ng/μL	–	40	–	–	–	–	yes
7	Plasmid-Cas9 / 1 μg/μL	sgtraEx1 / 1 μg/μL	–	40	–	–	–	–	yes
8	Plasmid-dCas9 / 1 μg/μL	sgtraEx1 / 1 μg/μL	–	40	–	–	–	–	yes
9	buffer	buffer	buffer	40	–	–	–	–	no
10	–	sgtraEx1 / 1 μg/μL	–	40	–	–	–	–	no
11	Cas9 Protein / 1,8 μg/μL	–	–	40	–	–	–	–	no
12	Plasmid-Cas9 / 1 μg/μL	–	–	40	–	–	–	–	no
13	Plasmid-dCas9 / 1 μg/μL	–	–	40	–	–	–	–	no

Although upon Cas9-sgtraEx1 RNP injections we observed a very limited survival rate, the XX adults displayed some interesting features (Table 1, set n. 2). Indeed, the G_0_ progeny was composed not only of six unaffected females, as we expected (Fig. 2 set 2), but also six XX males. These findings are suggestive of an unusually high rate of biallelic mutations. It is also worth noting that no intersexes were observed, suggesting an all-or-none effect on *Cctra* female-specific function of the Cas9 + sgtraEx1 injection.

Considering the apparently high efficiency of the ribonucleoprotein injections in set n. 2 (Table 1) in masculinizing 50% of the G_0_ XX individuals, we reasoned that 1) also the XX females contained at least some somatic clones bearing monoallelic *Cctra* mutations, though with no phenotypic effect, and that 2) a high mutagenic rate could also be present in the germ lines of these XX female adults as in the XX males. Assuming a 20–50% transmission rate of mutant alleles for each G_0_ parent to the next progeny as previously observed in the Medfly [16], the probability to observe a double mutant individual in the G_1_ progeny (Fig. 2) would be in a range of 4–25%. When we crossed among them the six XX males and six XX females from injection set n. 2 (Table 1), all 100 individuals of G_1_ were females, indicating that the six XX masculinized fathers were fertile. However, the absence of XX males in the G_1_ progeny indicated that, if any *Cctra* mutation was induced by Cas9 in the parental germ lines (male and female ones), the transmission rate was lower than 10% for each parent as the expected G_1_ heteroallelic mutants frequency would be less than 1% of the progeny (hence not detectable among a number of 100 individuals).

### Lack of indels in the targeted *Cctra* region in most cDNA clones from XX G_0_ males and in all genomic DNA clones from G_0_ XX females

The six females and six XX males, which composed the G_0_ progeny of set n. 2, were analyzed respectively by RT-PCR and genomic PCR, to investigate *Cctra* splicing and DNA sequence of the targeted site.

**Fig. 3:**
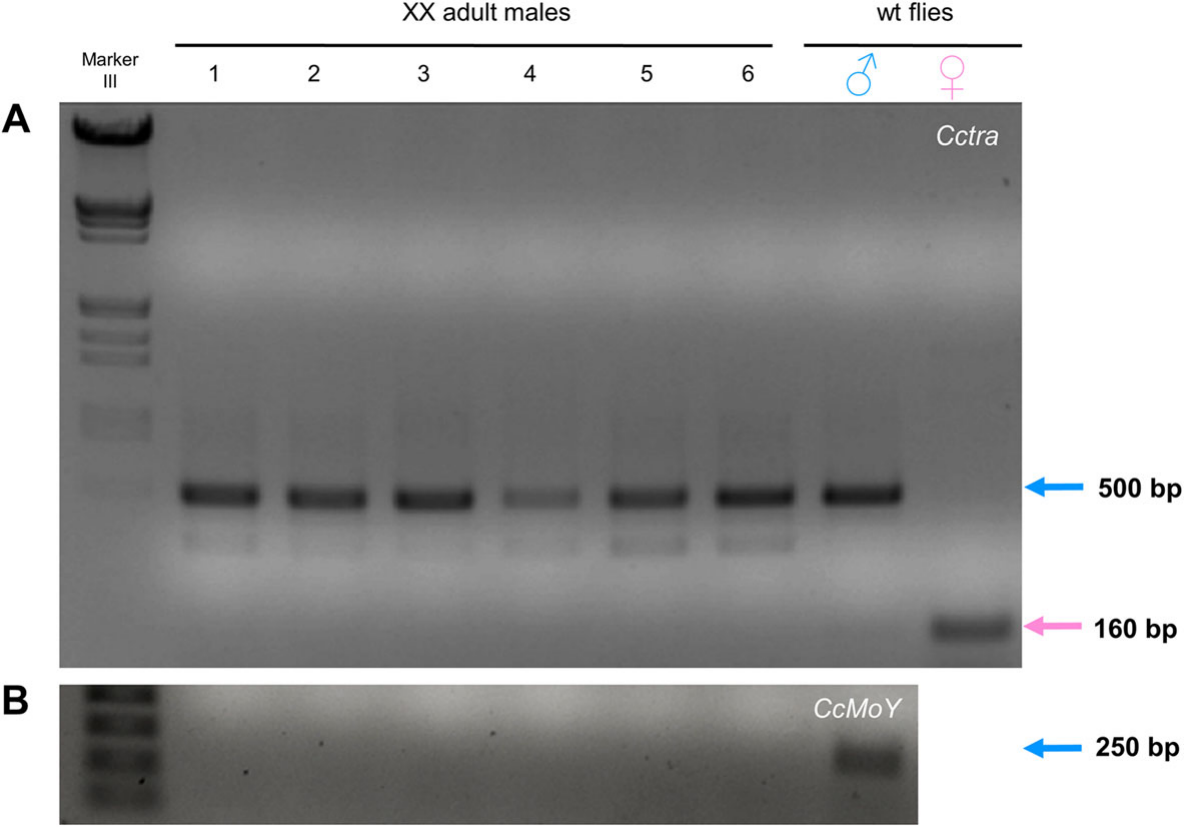
RT-PCR analysis of reverted XX males. **(A)** The six XX males from injection set n. 2 (Table 1) showed only male-specific *Cctra* transcripts (500 bp) and shorter less abundant splicing variants. No traces of female-specific transcripts were visible in the gel electrophoresis (160 bp) **(B)** RT-PCR with *MoY*-specific primers indirectly confirmed the absence of the Y chromosome in these six fully reverted XX males and its presence in XY flies (positive control)

The six reverted XX G_0_ males showed only male-specific *Cctra* transcripts, as expected for adult flies having a full fertile male phenotype (Fig. 3). These data, together with the absence of female-specific *Cctra* mRNAs in all six males, suggested the presence of biallelic mutations in most, if not all, of the somatic cells of these XX G_0_ males. Shotgun plasmid cloning of the RT-PCR reactions from the six XX G_0_ males, followed by PCR colony screening of 30 clones (five colonies A-E, for each of the six XX males) led us to arbitrarily select 13 clones for sequencing (two or three cDNA clones for each male) (Additional file 1: Figure S2 and Figure S3)(Additional file 2). The splicing isoforms detected in the six XX males corresponded mostly to the know M1 and M2 *Cctra* male-specific isoforms. Out of 13 cDNA clones from the six XX males, seven correspond to male-specific *Cctra* isoform M1 (59 aa), two correspond to the male-specific isoform M2 (99 aa), one to a new splicing male-specific isoform (Male 1D encoding a 44 aa long protein isoform, named M3), one to the female-specific isoform (429 aa) and one to an unspliced longer isoform (male 5C) (Additional file 1: Figure S3; Additional file 2) [6]. Very surprisingly, despite the observed *Cctra* male-specific full shift, 11 cDNA clones showed only wild-type sequences (Additional file 1: Figure S3). In one XX male, we have found a cDNA showing a 16 bp long deletion (cDNA 3E), in addition to a wild type cDNA clone (cDNA 3B) (Additional file 1: Figure S1D). The mutated cDNA encodes a 35 aa long CcTRA truncated protein. Another XX male contained two wild type (5B and 5D) cDNA clones and a mutated one lacking of 5 bp (5C) (Additional file 1: Figure S1C). This cDNA Male 5C encoded a truncated CcTRA protein of only 42 aa (Additional file 1: Figure S1C). The male 5C cDNA from unspliced *Cctra* RNA seems to correspond to a previously described RNA only present in adult females [6]. The female specificity of this unspliced product could be due to the binding of female-specific CcTRA/CcTRA-2 complex to the *Cctra* pre-mRNA required for the autoregulation. Interestingly, the *Cctra* unspliced isoform we found in one XX male contains also the Cas9-induced 5 bp deletion. We speculated that the 5 bp long deleted region of *Cctra* could be involved in enhancing the recognition of the 5′ donor site, which is only 20 bp downstream to the deletion by the spliceosome. We have found that these 5 bp are perfectly conserved in other Tephritidae *tra* orthologues, suggesting their requirement for proper *Cctra* male-specific default splicing (Additional file 1: Figure S1E). On the other hand, the cDNA 3E containing even a larger deletion (16 bp) of the same *Cctra* region performed a male-specific splicing, indicating that this sequence (14 bp out of 20 bp targeted sequence conserved in 5 distantly related Tephritidae species; Additional file 1: Figure S1E) is not strictly required to perform this alternative splicing but could be involved in performing the female-specific one. Collectively, these data showed intriguingly that the six XX males had a full switch from female-specific to male-specific splicing of *Cctra*, even in the presence of a very low (hence, mostly monoallelic) or even zero number of mutant *Cctra* alleles.

As the six XX females showed a normal female phenotype, female-specific *Cctra* splicing was expected to be found, even if they were carrying monoallelic *Cctra* mutations (Fig. 2). Hence, *Cctra* genomic DNA (rather than RNA or cDNA) was analyzed and eight plasmid clones showed only *Cctra* wild-type sequences (Additional file 1: Figure S4; Additional file 3). The lack of *Cctra* mutations in the DNA clones from all six G_0_ females and in most of the cDNA from the six XX males is consistent with the absence of XX mutant heteroallelic *Cctra*^*− 1*^/*Cctra*^*− 2*^ males we observed in their G_1_ progeny.

### Embryos co-injections of dCas9 and sgtraEx1 lead to partially masculinized XX embryos and adult flies, indicating a long-lasting effect in the absence of mutations

**Fig. 4:**
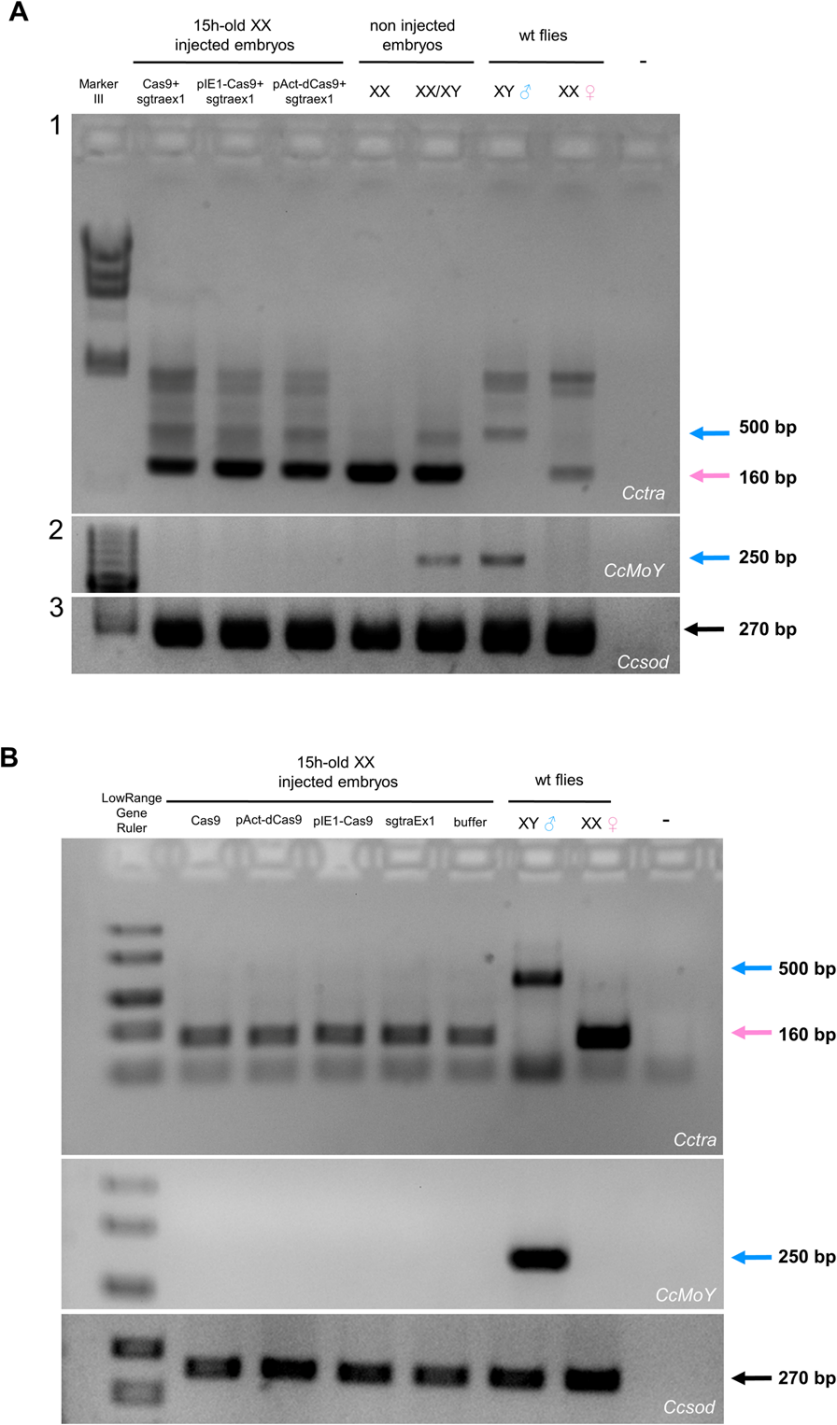
Molecular partial masculinization of XX embryos by dCas9 targeting *Cctra.* **(A)** RT-PCR analysis of *Cctra* sex-specific transcripts in 15 h old XX embryos, following injections of various samples (Table 1, set n. 6–8) at 1 h after egg laying (Panel 1). The XX embryos were injected respectively with Cas9 protein+sgtraEx1 (first lane), with sgtraEx1 + Cas9-encoding plasmid (pIE1-Cas9; second lane) and with dCas9-encoding plasmid (pAct-dCas9) (third lane). Male-specific *Cctra* transcripts were detected (500 bp cDNA fragment) in all three samples of injected XX embryos, in addition to the female-specific transcripts (160 bp cDNA fragment). RT-PCR analysis of the Y-linked *MoY* gene indirectly confirmed the absence of Y chromosome in all 3 samples and in XX females, and its presence in a mixed XX/XY embryos sample and in adult XY flies (Panel 2). *CcSOD* positive control is shown in panel 3. **(B)** Negative controls. RT-PCR of *Cctra* sex-specific transcripts in 15 h old XX embryos, following injections at 1 h after egg laying (Table 1, set n. 9–13). The XX embryos were injected respectively with Cas9 protein, Cas9-encoding plasmid, dCas9-encoding plasmid, with sgtraEx1 and with buffer alone. Only female-specific *Cctra* transcripts were detected (160 bp) in all samples of injected XX embryos (Panel 1). In Panel 2 and 3, the control of the karyotype and positive controls, conducted as in A, are shown

We speculated that this Cas9 ribonucleoprotein complex was able to bind but not efficiently cut the *Cctra* DNA target site. The dead Cas9 (dCas9) is a mutant Cas9 which lacks of only the endonuclease activity and it can be used to perform transient transcriptional repression, named CRISPR interference, or iCRISPR [23–25]. We co-injected the sgRNA sgtraEx1 (1 μg/μl) with a plasmid bearing a dCas9 transgene under a *Drosophila* actin promoter, expecting that dCas9 would be produced after transcription and translation and would bind the available sgRNA [24] (Table 1, set n. 5). A higher concentration of sgRNA (1 μg/μl; see mat. & meth.) was used in the injected mix because we expected that these RNA molecules being not pre-assembled with Cas9 before injections, could have been more exposed to degradation. This high concentration of sgRNA alone had no effect on *Cctra* female-specific splicing in injected XX embryos and in developed XX adults (Table 1, set n. 10; Fig. 4, B). Three out of ten XX adult females showed malformations of the gonadal apparatus, which suggested a mild masculinization effect (Additional file 1: Figure S5A). RT-PCR analysis of *Cctra* on RNA from these three XX adult flies confirmed the presence of also male-specific RNAs, indicating that the ovipositor malformations are likely the result of a partial masculinization during development (Additional file 1: Figure S5B). These data indicated that the dCas9 can induce a partial masculinization of XX embryos and a stable shift toward male-specific splicing of *Cctra* likely in some somatic clones.

We investigated the sex-specific splicing pattern of *Cctra* in XX-only embryos, injected at 1 h after egg laying, and developed for additional 14 h (set n. 6–13). Injections of Cas9 recombinant protein+sgtraEx1 RNP (set n. 6), or co-injections of sgtraEx1 with plasmids encoding either Cas9 (driven by E1 early promoter [23]) or dCas9 (set n. 7 and set n. 8) led to the appearance of additional male-specific *Cctra* transcripts (Table 1, Fig. 4A). Injections of only buffer, sgtraEx1, Cas9 protein, Cas9-encoding and dCas9-encoding plasmids had no effect (Fig. 4B). These data suggest that, as Cas9, also dCas9 can induce male-specific splicing of *Cctra* during the first hours of development. Hence, no *Cctra* mutations are required for this splicing change.

## Discussion

The Cas9-sgtraEx1 RNPs injected into XX Medfly embryos led to a masculinization of 50% of XX individuals. All six XX males showed by RT-PCR and cDNA sequencing analyses mostly male-specific *Cctra* isoforms, concordant with the observed male phenotype. Unexpectedly, no indel mutations were detected in cDNA fragments from the Cas9 targeted site in four out of six males. Two XX males showed a mix of wild type and mutated cDNA fragments, suggesting the presence of monoallelic mutations in some cellular regions. XX males and XX females from injected embryos produced a G_1_ consisting of only XX females, which indicated absence of homozygous individuals for mutant *Cctra* alleles. We concluded that very low mutation rate, if any, was reached not only in the soma but also in the germ line of injected G_0_ individuals. In contrast, Meccariello et al. [16], observed that mutant mosaic G_0_ flies mutagenized by Cas9 in the *white-eye* gene transmitted germ line mutations to the progeny at a very high rate (up to 100%). Hence, the Cas9-sgtraEx1 is likely very inefficient in mediating Cas9 gene disruption but surprisingly very efficient in masculinizing XX individuals. These data suggested that the Cas9 ribonucleoprotein promoted masculinization by inducing male-specific *Cctra* splicing in 50% of XX embryos which developed into adults by a mechanism different than gene disruption.

We reasoned that the sgRNA designed for the CRISPR experiment and/or the *Cctra* targeted region structural features posed limitations in the second step of the Cas9 action, namely the endonuclease activity, but not in the first step, the binding to the *Cctra* genetic locus. Indeed, a two-state model for Cas9 binding and cleavage was recently proposed: a seed match triggers binding but only extensive pairing with target DNA leads to cleavage [26]. Target sequence mismatches can induce the Cas9-RNP complex to bind also off-target sites without DNA cleavage, because the transition to the active conformation is prevented [26, 27]. Co-injections of sgtraEx1 and a plasmid encoding dCas9 into XX embryos induced a partial shift of *Cctra* splicing toward male-specific pattern after few hours of development and the development of XX females (three out of ten) with malformed ovipositor. These females were found to be molecular intersexes, as they showed both female-specific and male-specific *Cctra* isoforms. Also, these malformed females showed a mix of male and female-specific *Cctra* transcripts and, hence, correspond to partially masculinized individuals (intersexes) [6]. Similar malformed ovipositors were observed by Pane et al. [6] following embryonic RNA against *Cctra*. The lack of fully masculinized XX individuals in the progeny of ten individuals suggests a reduced efficiency of dCas9 in masculinizing XX individuals. However, this could be due to the different delivery methods of Cas9 and dCas9. In the first case, purified recombinant Cas9 ready to act was injected, while in the second case a dCas9 encoding transgene was transcribed from a *Drosophila* actin promoter, after embryos injections of a plasmid. Lower efficiency is to be expected in the second delivery method, due to the transcription and translation steps required to express dCas9. The transient binding of Cas9 RNPs to the 5′ *Cctra* DNA region could have reduced the *Cctra* zygotic transcription during the first hours of embryogenesis and, hence, the accumulation of female specifically spliced *Cctra* mRNA, promoted by the maternal *Cctra* contribution in XX embryos. This transient reduction of newly transcribed *Cctra* female-specific mRNA and of the encoded CcTRA protein in the XX embryos could have blocked the establishment of *Cctra* female-specific autoregulation leading to a negative epigenetic effect on *Cctra*. Similarly, a transient depletion of *Cctra* mRNA by embryonic RNAi led to a collapse of its positive autoregulation and to obtain XX fertile males expressing male-specific *Cctra* RNAs [6].

## Conclusions

The question if CcTRA male-specific ORFs are required for male-specific *Cctra* splicing remains still open, as biallelic *Cctra* mutant XX males were not obtained. The second question if the chosen sgtraEx1 guide RNA is suitable for future gene drive strategy aimed to efficient mutagenesis by Cas9 had a conclusive and negative reply, as very low mutagenesis rate was observed. However, these data support the hypothesis that the transient binding of the Cas9-sgtraEx1 ribonucleoprotein complex on the first *Cctra* exon during the first hours of embryogenesis led to a repression of the establishment of the *Cctra* female-specific autoregulation in XX embryos even in the absence of induced mutations. Our study raises new general issues concerning the use of CRISPR/Cas9 method. We serendipitously uncovered a novel problem of unplanned stable changes in the expression of genes able to autoregulate, which calls for further investigation. If a Cas9 + sgRNA binds to off target sequences of autoregulating bistable genes, this event can provoke long lasting epigenetic effects even in the absence of DNA mutations.

## Methods

### Rearing of *Ceratitis capitata*

Wild type (WT) and transgenic Medfly lines were maintained under standard rearing conditions. The WT *Benakeion* strain, which has been reared in laboratories for more than 20 years, was obtained from P. A. Mourikis 30 years ago (Benakeion Institute of Phytopathology, Athens, Greece). The strains were reared in standard laboratory conditions at 25 °C, 70% relative humidity and 12:12 h light–dark regimen. Adult flies were fed yeast/sucrose powder (1:2).

### RNP complex assembly and injections

Cas9 was expressed as his-tagged protein and purified from bacteria [16, 19]. sgRNA was designed using the CHOPCHOP online software [28]. The lack of SNPs within this 20 nt long sequence in three different *Ceratitis* lines (Benakeion, used in this study, ISPRA [22] and FAM18 *Ceratitis* [8]) suggested that no Cas9-resistant *Cctra* alleles would be already present in individuals of these lab strains. Template for sgRNA in vitro transcription were generated by annealing two complementary oligonucleotides (PAGE-purified, Life Technologies) as previously described [16, 21], using the primers F-sgtraEx1 and Reverse-Crispr from Life Technologies (Additional file 4). sgRNA was synthesized according to instructions of the Megascript® T7 kit (Ambion) with 1 μg of DNA template and a 5′ flanking T7 promoter. After RNA synthesis, the template was removed by incubating with TurboDNase® (Ambion) for 15 min at 37°. Prior to the injection, the RNP complex was prepared by mixing 1.8 μg/μL of purified Cas9 protein with approximately 200 ng/μL of sgtraEx1, containing 300 mM KCl [16]. The mix was incubated for 10 min at 37 °C. A glass needle was filled with the pre-loaded sgtraEx1-Cas9 mix and the injection was performed into the posterior end of embryos collected 45 min after egg laying as described for RNA interference in *Ceratitis capitata* [6]. When injecting sgRNA alone, a 5 times higher concentration (1 μg/μL) was used in the injected mix, because we expected that these RNA molecules, being not pre-assembled with Cas9 before injections, could have been more exposed to degradation.

### RNAi and XX-only progeny production

A *Cctra c*DNA 800 bp long fragment was PCR amplified using RNA from female adults of *C. capitata* and longer 164+/900- primers, introducing a T7 promoter sequence at each extremity. In vitro transcription of *Cctra* dsRNA was performed using the Ambion MEGAscript® RNAi kit T7 RNA polymerase, following manufacturer instructions. Embryonic RNAi (0.5 μg/μL dsRNA solution) was used to repress *Cctra* in XX/XY embryos and to produce male only progeny. Single males from this progeny were crossed with three females in small cages and the crosses having XX males were identify by Y-specific PCR (Y-specific primers) [8] on a small sample of laid embryos.

### dCas9 encoding plasmid and injections

Plasmid expressing dCas9 under the control of the *Drosophila melanogaster* actin promoter was kindly provided to GS by Lenny Rabinow (Perrimon’s lab, Harvard, USA) [23]. A mix containing 1 μg/μL of pAct-dCas9 plasmid and 1 μg/μL of sgtraEx1 transcribed in vitro into the posterior end of embryos. We used 5 times higher concentration of sgRNA because we expected that these small RNA molecules being not pre-assembled with Cas9 before injections, could have been more exposed to degradation.

### RNA extraction and RT-PCR

Total RNA was extracted from pools of embryos, intersexes, male and female adults using TRIzol® Reagent (Invitrogen®) following manufacturer instructions. Oligo-dT-primed cDNA was prepared from DNAse-treated total RNA using EuroScript® m-MLV reverse transcriptase (Euroclone®). RT-PCR expression analysis was performed with the following primers: *Cctra* 164+/320-, *CcSOD+/CcSOD-* and *CcMoY A+/CcMoY A-* (Additional file 4). For XX malformed females obtained from XX only embryos with dCas9 and sgtraEx1 *Cctra* RT-PCR was performed using *Cctra* 164+/900- primers pair, annealing on the same exon as *Cctra* 320- primer (Additional file 4). Gel electrophoresis diagnostic amplicon run was performed using Marker III (Lambda genomic DNA digested with EcoRI/HindIII) or 100 bp ladder from Thermo Scientific®.

### DNA extraction and molecular analysis

DNA extraction was performed, with minor modifications, according to the protocol of Holmes and Bonner et al. [29]. Adult XX female flies G_0_ were placed in a 1.5 ml tube and manually crushed with a pestle in 200 ml Holmes Bonner buffer (Urea 7 M, 281 Tris-HCl 100 mM pH 8.0, EDTA 10 mM pH 8.0, NaCl 350 mM, SDS 2%). Subsequently, DNA was purified by phenol/chloroform extraction, followed by chloroform extraction and ethanol precipitation. The pellet was resuspended in 30 μl water containing RNase A.

### cDNA and gDNA cloning and sequencing

PCR cDNA fragments from XX adult males were cloned into pGEM-T Easy Promega® vector according manufacturer instruction. PCR colony screening was carried out using 164+ and 320- primers. Positive colonies were used to extract plasmid DNA which was sequenced using Applied Biosystem® Big Dye v 3.1. Genomic DNA from the six G_0_ XX females was used as template to amplify the region encompassing the target sites, using the primers *Cctra* 164+ and *Cctra* 164-Rev (Additional file 4). DreamTaq (Life Technologies) polymerase was used for PCR amplifications according to the manufacturer’s instructions. The PCR products were purified with StrataPrep PCR Purification Kit (Agilent Technologies) and subcloned using StrataClone PCR cloning Kit (Agilent Technologies). Positive clones were sequenced by Sanger method and ABI 310 Automated Sequencer (Applied Biosystems) using the primer *Cctra* 164+ (Additional file 4).

### RNA extraction from injected embryos after 15 h of development

The pools of 40 embryos injected with various mixes (Table 1) were let develop for 15 h at 25 °C, 70% relative humidity. The embryos were then detached from the cover slip using heptane, which dissolves the glue, and collected in a 1.5 mL tube. They were then washed three times with 1X PBS to remove heptane before RNA extraction was performed using TRIzol® Reagent (Invitrogen®) following manufacturer instructions. The RNA samples were analyzed for *Cctra* splicing pattern by RT-PCR using the primers *Cctra* 164+ and *Cctra* 900- (Additional file 4).). The cDNA and genomic sequences were deposited at the GenBank database with the following accession numbers: MW200161 to MW200180.

## Supplementary Information

**Additional file 1.**

**Additional file 2.**

**Additional file 3.**

**Additional file 4.**

**Additional file 5.**

## Data Availability

The cDNA and genomic sequences were deposited at the GenBank database with the following accession numbers: MW200161 to MW200180.

## Abbreviations

*SIT*: Sterile Insect Technique
*ORF*: Open Reading Frame
*CRISPR*: Clustered Regulated InterSpaced Palindromic Repeats
*RNP*: Ribo-Nucleo-Proteic Complex
*dsRNA*: double strand RNA
*sgRNA*: single guide RNA
*iCRISPR*: interference CRISPR
*dCas9*: dead Cas9
*SNP*: Single Nucleotide Polymorphism
*RNAi*: RNA interference
*WT*: Wild-Type
