# Supplementary material for: Targeting the autosomal Ceratitis capitata transformer gene using Cas9 or dCas9 to masculinize XX individuals without inducing mutations
Source: BMC Genet. 2020 Dec 18;21(Suppl 2):150. doi: 10.1186/s12863-020-00941-4 (PMC7747381; doi:10.1186/s12863-020-00941-4)
Supplement: Supplementary file 1 — Additional file 1. [file 12863_2020_941_MOESM1_ESM.pdf]

Supplementary figures for:

Targeting the autosomal *Ceratitis capitata transformer* gene using Cas9 or dCas9 to masculinize XX individuals without inducing mutations.

Pasquale Primo[1*], Angela Meccariello[1*], Maria Grazia Inghilterra[1], Andrea Gravina[1], Giuseppe Del Corsano[1], Gennaro Volpe[1], Germano Sollazzo[1], Serena Aceto[1], Mark D. Robinson[2], Marco Salvemini[1] and Giuseppe Saccone[1]

[1]Department of Biology, University of Naples Federico II, 80126, Naples, Italy.
[2]Department of Molecular Life Sciences and SIB Swiss Institute of Bioinformatics, University of Zurich, Winterthurerstrasse 190, CH-8057, Zurich, Switzerland.

*equal contribution

Correspondence:
giuseppe.saccone@unina.it,
Department of Biology, University of Naples “Federico II”,
80126, Naples, Italy

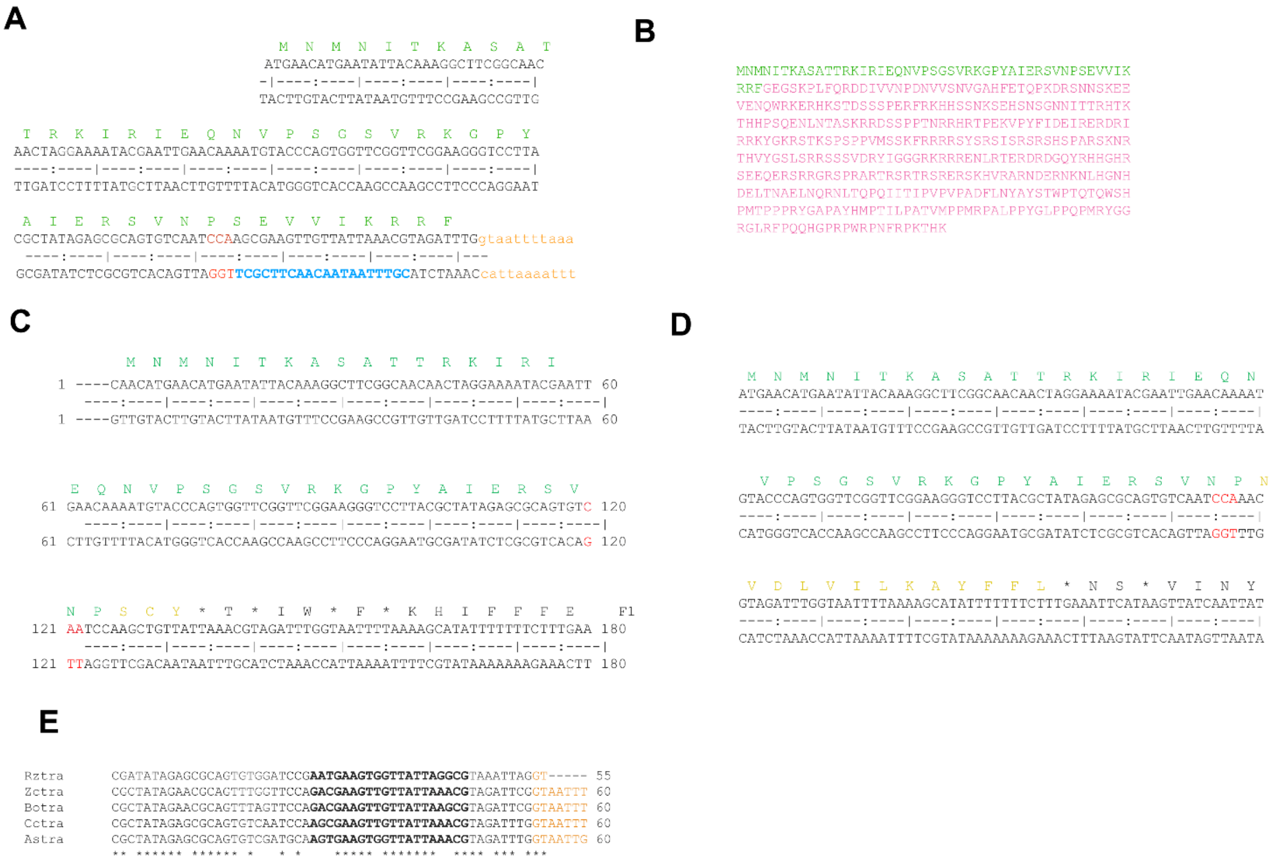

Figure S1 ***Cctra*** **nucleotide and amino acid sequences of the targeted region. (A)** The *Cctra* genomic sequence corresponding to the first exon and the CcTRA amino acid sequence (in green) are shown. In lower case/orange, the intronic region is shown. The traEx1 sgRNA target site (antisense) is indicated in blue and the PAM (TGG) in red. **(B)** The CcTRA female-specific amino acid sequence is shown. Frameshift mutations cause the loss of the protein region indicated in pink. **(C)** In Male 3E cDNA, a 16 bp deletion (in the region following the PAM indicated in red) led to a truncated 35 aa long CcTRA putative protein **(D)** In Male 5C cDNA, a 5 bp deletion (in the region following the PAM indicated in red) led to a truncated 42 aa long CcTRA putative protein. **(E)** Clustal sequence alignment of *tra* exon 1 homologous regions from *Rhagoletis zephyria*, *Zeugodacus cucurbitae*, *Bactrocera oleae*, *Ceratitis capitata* and *Anastrepha suspensa*. In bold black the *Cctra* Cas9 targeted region and in bold the PAM sequence (antisense strand).

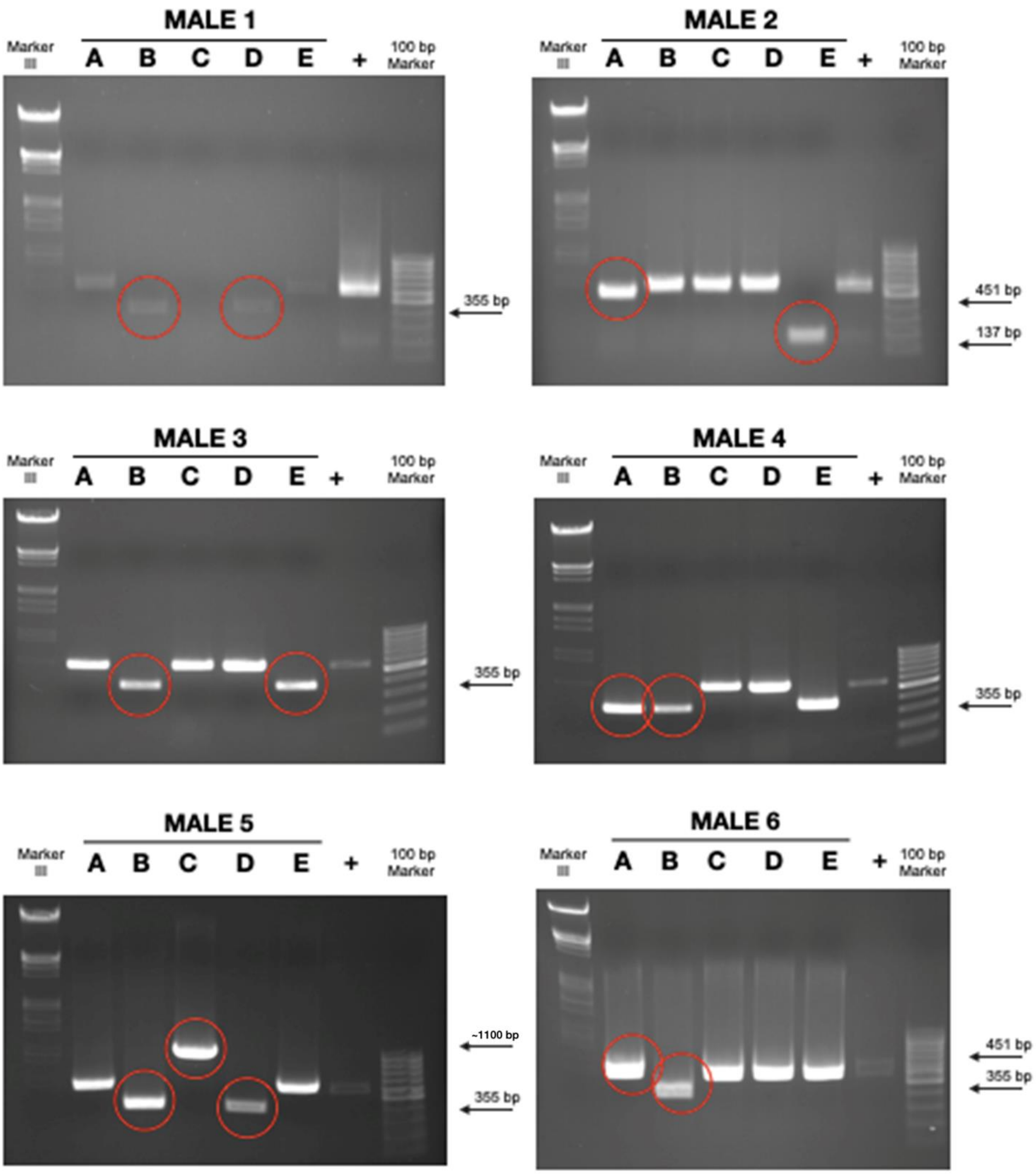

Figure S2 PCR screening of bacterial colonies following shotgun cloning of *Cctra* cDNA products from the six XX G_0 males. Six gel electrophoreses are shown, each corresponding to one of the six XX males. PCR was performed using 164+/320- primers on five colonies diluted in 10 microliters of water, from each of the six agar plates, which resulted from the shotgun cloning and bacterial transformation. The two (or three) clones for each male selected for DNA sequencing are indicated by a red circle.

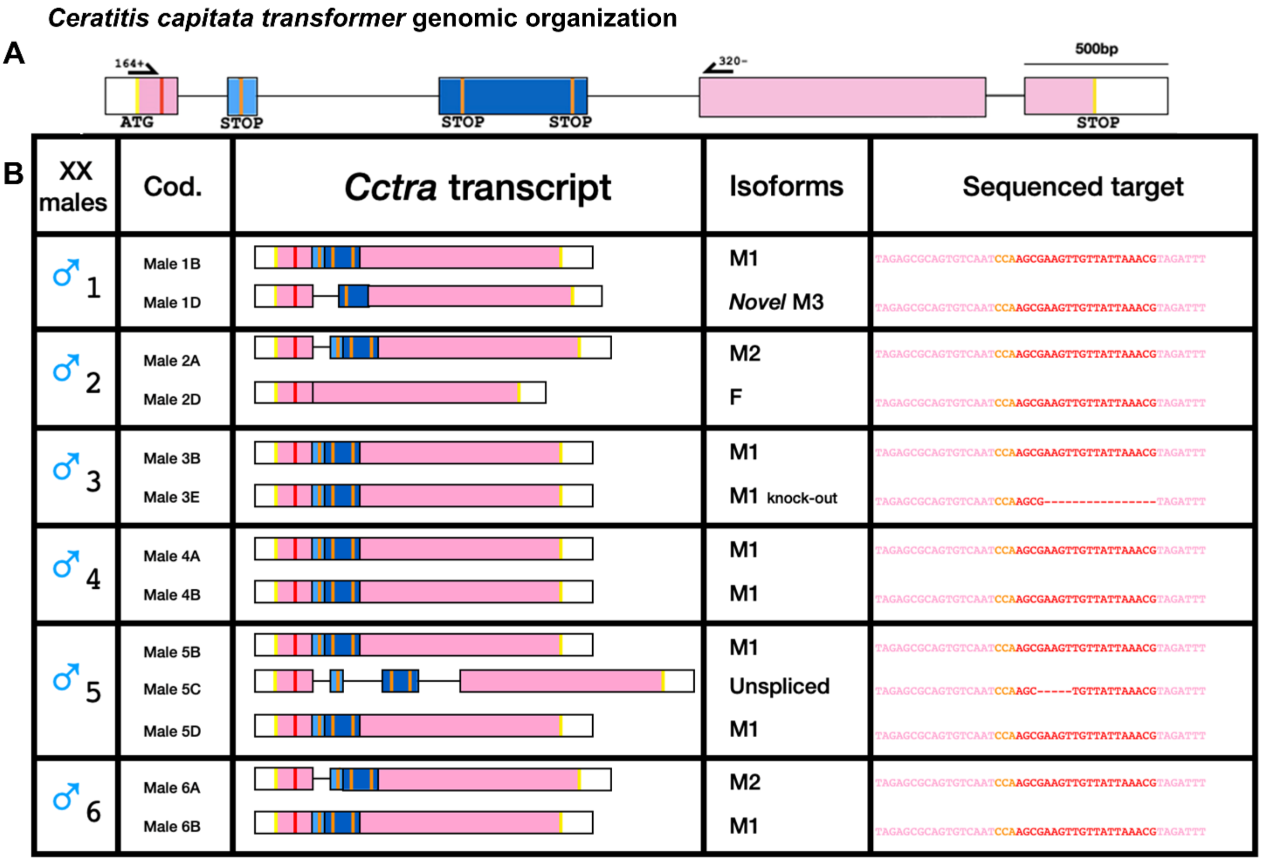

Figure S3 XX reverted males produce mainly male-specific *Cctra* transcripts showing wild type sequence in the targeted site. (A) *Cctra* locus consists of male-specific exons (light blue and blue), introducing stop codons in the ORF and of a female-specific long ORF (pink) (see also Fig. 1). **(B)** This scheme shows the four types *Cctra* splicing isoforms detected in the 13 cDNA cloned fragments from six XX G_0 males (Additional file 1: Figure S3). Two cDNA clones were sequenced and analyzed from each adult (three cDNA clones for the male n. 5). Eight of 13 cDNAs corresponded to the *Cctra* male-specific isoform M1 (Fig. 1). Two corresponded to the *Cctra* male-specific isoform M2. One cDNA corresponded to the female-specific cDNA product (Additional file 1: Figure S2, shorter PCR product D in Male 2; cDNA 2D). An unspliced *Cctra* cDNA product (cDNA 5C) was obtained from XX male n. 5 (Additional file 1: Figure S1D). Two males (n. 3 and 5) contained two independent *Cctra* mutations (deletions) in the targeted site, together with wild-type cDNA sequences. In five out of six XX males, a mix of different splicing variants or of mutant wild type sequence were detected.

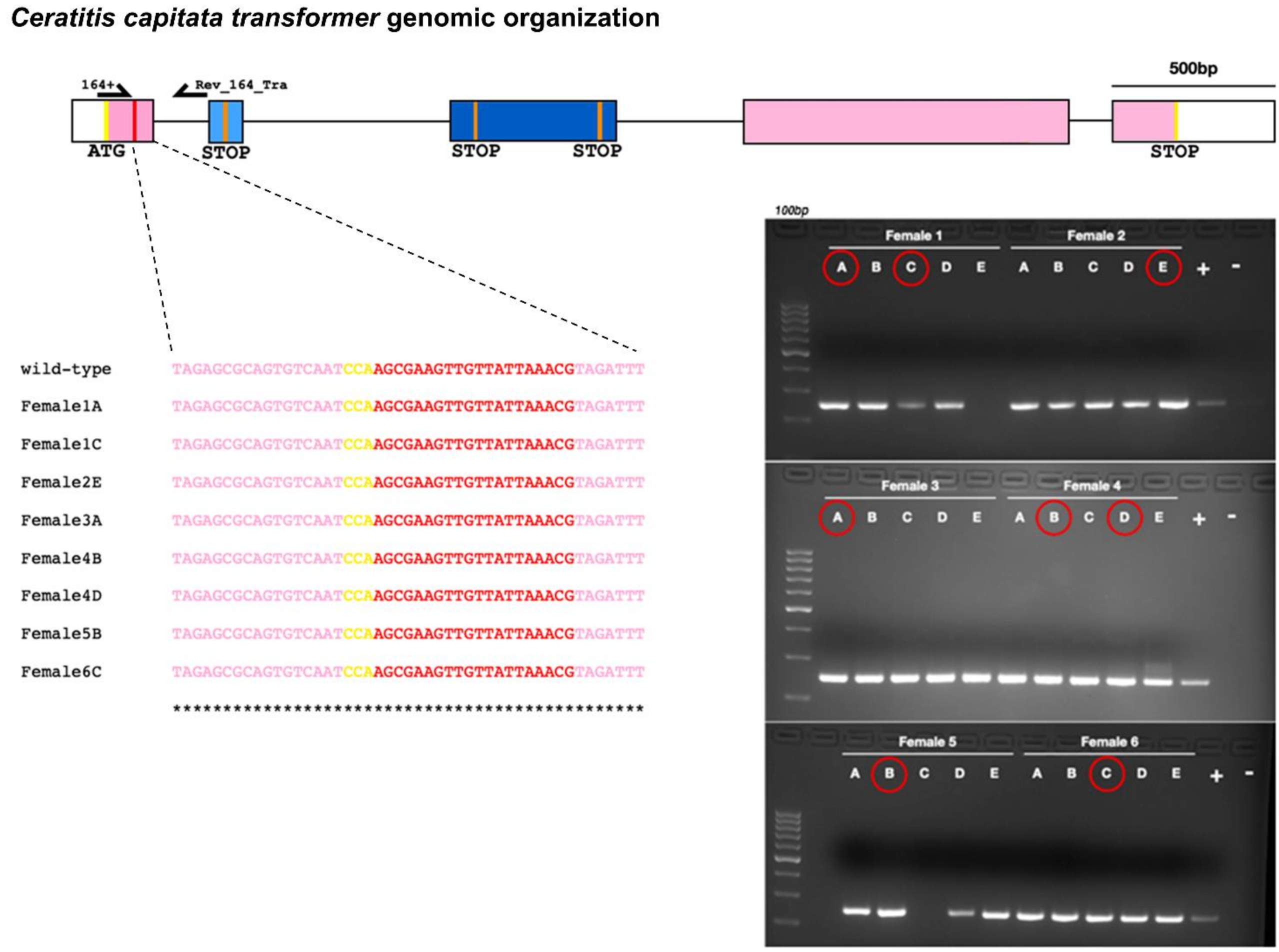

Figure S4 PCR screening of bacterial colonies following shotgun cloning of *Cctra* genomic DNA products from the six XX G_0 females.

Genomic DNA was extracted from each of the six females (Table 1, set 4) and the targeted *Cctra* region was amplified by PCR. Shotgun cloning and bacterial transformation led to obtain plasmid clones. Six gel electrophoreses are shown, corresponding to the six XX females. Screening of bacterial colonies were performed on five randomly chosen colonies from each of six agar plates (one for each female). *Cctra* gDNA clones for each female selected for DNA sequencing are indicated by a red circle. Eight genomic DNA clones showed wild type *Cctra* sequence in the targeted site.

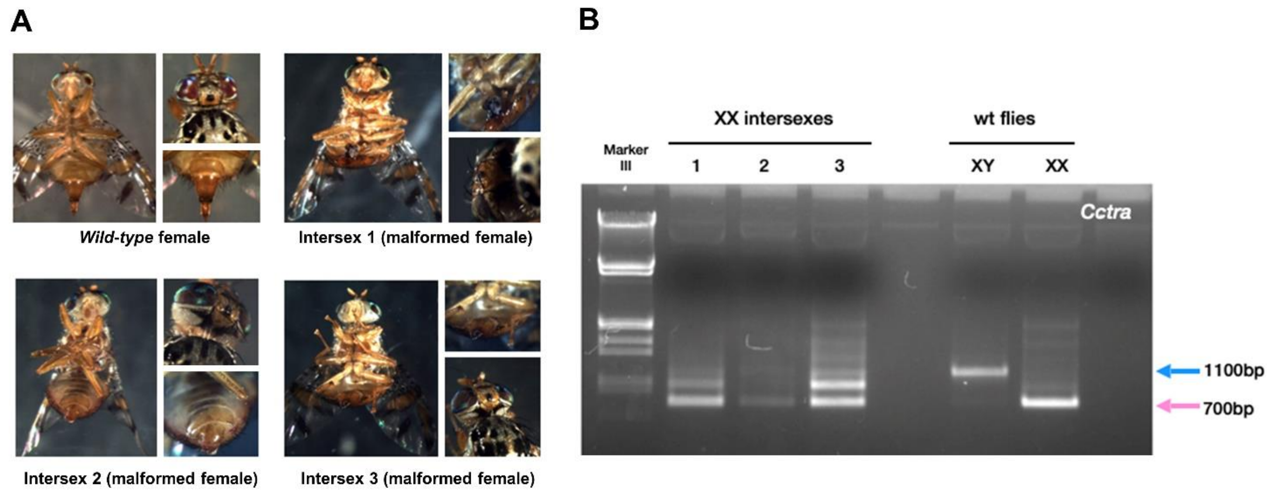

Figure S5 Transient embryonic CRISPR interference by a dCas9 induced ovipositor malformations and partial molecular masculinization in XX individuals. **(A)** Three adult females (Table 1, injection set n. 5) showed malformations of the ovipositor and **(B)** a mix of male-specific (1100 bp long cDNA fragment) and female-specific (700 bp long cDNA fragment) indicating partial masculinization and hence intersexuality.
