# Supplementary material for: Targeting the autosomal Ceratitis capitata transformer gene using Cas9 or dCas9 to masculinize XX individuals without inducing mutations
Source: BMC Genet. 2020 Dec 18;21(Suppl 2):150. doi: 10.1186/s12863-020-00941-4 (PMC7747381; doi:10.1186/s12863-020-00941-4)
Supplement: Supplementary file 2 — Additional file 2. [file 12863_2020_941_MOESM2_ESM.pdf]

Additional file n. 2

13 cDNA clone sequences from six XX males. The PAM sequence (antisense) is indicated in red bold.

Male 1B
TATAGAGCGCAGTGTCAAT**CCA**AGCGAAGTTGTTATTAAACGTAGATTTGGTAATTTTAAAAGCATATTTTTT
TCTTTGAAATTCATAAGTTATCAATTATCGATGGAAATGTATTCTATGGAGAACGTTTTACCCGATGAATGGG
TGCAAAAATTATTTTACCTTCAAATCTACAATCAACACACGCTAACTTTTGTGACTTGATCAACTCTCACCTG
GAAAAGCAACCAACTACAATCAACATTCTATGGGATAATCGACAATGTGAAGGCTCAAAGCCTCTGTTTCAAC
GCGACGACATCGTTGTAAACCCTGATAATGTTGTAAGTAATGTGGGTGCTCATTTTGAAA

Male 1D (Novel M3 isoform)
TATAGAGCGCAGTGTCAAT**CCA**AGCGAAGTTGTTATTAAACGTAGATTTGTAAACATATAACGATTGTCTATG
TAATAAGTAAACCAAGGAAAGGGAAAATATCTACAATCAACAAGCCATTGTTGCAGCAACAAAGCAACTGAAA
CTACAATCAACATTCAATAAACTTGGGTAATTTGGAATTTAATTCTCTGGGACACCTGTGGATTACAACAATC
AACTCGAAACTTATTATACAATGTGAGGGCTCAAAGCCTCTGTTTCAACGCGACGACATCGTTGTAAACCCTG
ATAATGTTGAAAGTAATGTGGGTGCTCATTTTGAAA

Novel male-specific CcTRA isoform M3 (44aa):
MNMNITKASATTRKIRIEQNVPSGSVRKGPYAIERSRQSKRSCY

Male 2A
TATAGAGCGCAGTGTCAAT**CCA**AGCGAAGTTGTTATTAAACGTAGATTTGAACGTTTTACCCGATGAATGGGT
GCAAAAATTATTTTACCTTCAAATCTACAATCAACACACGCTAACTTTTGTGACTTGATCAACTCTCACCTGG
AAAAGCAACCAACTACAATCAACATTCTATGGGATAATCGACAATCTATGTAATAAGTAAACCAAGGAAAGGG
AAAATATCTACAATCAACAAGCCATTGTTGCAGCAACAAAGCAACTGAAACTACAATCAACATTCAATAAACT
TGGGTAATTTGGAATTTAATTCTCTGGGACACCTGTGGATTACAACAATCAACTCGAAACTTATTATACAATG
TGAAGGCTCAAAGCCTCTGTTTCAACGCGACGACATCGTTGTAAACCCTGATAATGTTGTAAGTAATGTGGGT
GCTCATTTTGAAA

Male 2D (female-specific isoform)
TATAGAGCGCAGTGTCAAT**CCA**AGCGAAGTTGTTATTAAACGTAGATTTGGTGAAGGCTCAAAGCCTCTGTTT
CAACGCGACGACATCGTTGTAAACCCTGATAATGTTGTAAGTAATGTGGGTGCTCATTTTGAAA

Male 3B
TATAGAGCGCAGTGTCAAT**CCA**AGCGAAGTTGTTATTAAACGTAGATTTGGTAATTTTAAAAGCATATTTTTT
TCTTTGAAATTCATAAGTTATCAATTATCGATGGAAATGTATTCTATGGAGAACGTTTTACCCGATGAATGGG
TGCAAAAATTATTTTACCTTCAAATCTACAATCAACACACGCTAACTTTTGTGACTTGATCAACTCTCACCTG
GAAAAGCAACCAACTACAATCAACATTCTATGGGATAATCGACAATGTGAAGGCTCAAAGCCTCTGTTTCAAC
GCGACGACATCGTTGTAAACCCTGATAATGTTGTAAGTAATGTGGGTGCTCATTTTGAAA

Male 3E (16 bp deletion between black/blue positions in bold)
TATAGAGCGCAGTGTCAAT**CCA**A**ACGTAG**ATTTGGTAATTTTAAAAGCATATTTTTTTCTTTGAAATTCATAA
GTTATCAATTATCGATGGAAATGTATTCTATGGAGAACGTTTTACCCGATGAATGGGTGCAAAAATTATTTTA
CCTTCAAATCTACAATCAACACACGCTAACTTTTGTGACTTGATCAACTCTCACCTGGAAAAGCAACCAACTA
CAATCAACATTCTATGGGATAATCGACAATGTGAAGGCTCAAAGCCTCTGTTTCAACGCGACGACATCGTTGT
AAACCCTGATAATGTTGTAAGTAATGTGGGTGCTCATTTTGAAA

Truncated CcTRA isoform encoded by this Male 3E cDNA (35 aa) from an adult XX male showing also wild-type cDNA clones, hence being a mosaic:
MNMNITKASATTRKIRIEQNVPNVDLVILKAYFFL

Male 4A
TATAGAGCGCAGTGTCAAT**CCA**AGCGAAGTTGTTATTAAACGTAGATTTGGTAATTTTAAAAGCATATTTTTT
TCTTTGAAATTCATAAGTTATCAATTATCGATGGAAATGTATTCTATGGAGAACGTTTTACCCGATGAATGGG
TGCAAAAATTATTTTACCTTCAAATCTACAATCAACACACGCTAACTTTTGTGACTTGATCAACTCTCACCTG
GAAAAGCAACCAACTACAATCAACATTCTATGGGATAATCGACAATGTGAAGGCTCAAAGCCTCTGTTTCAAC
GCGACGACATCGTTGTAAACCCTGATAATGTTGTAAGTAATGTGGGTGCTCATTTTGAAAT

Male 4B
TATAGAGCGCAGTGTCAAT**CCA**AGCGAAGTTGTTATTAAACGTAGATTTGGTAATTTTAAAAGCATATTTTTT
TCTTTGAAATTCATAAGTTATCAATTATCGATGGAAATGTATTCTATGGAGAACGTTTTACCCGATGAATGGG
TGCAAAAATTATTTTACCTTCAAATCTACAATCAACACACGCTAACTTTTGTGACTTGATCAACTCTCACCTG
GAAAAGCAACCAACTACAATCAACATTCTATGGGATAATCGACAATGTGAAGGCTCAAAGCCTCTGTTTCAAC
GCGACGACATCGTTGTAAACCCTGATAATGTTGTAAGTAATGTGGGTGCTCATTTTGAAA

Male 5B
TATAGAGCGCAGTGTCAAT**CCA**AGCGAAGTTGTTATTAAACGTAGATTTGGTAATTTTAAAAGCATATTTTTT
TCTTTGAAATTCATAAGTTATCAATTATCGATGGAAATGTATTCTATGGAGAACGTTTTACCCGATGAATGGG
TGCAAAAATTATTTTACCTTCAAATCTACAATCAACACACGCTAACTTTTGTGACTTGATCAACTCTCACCTG
GAAAAGCAACCAACTACAATCAACATTCTATGGGATAATCGACAATGTGAAGGCTCAAAGCCTCTGTTTCAAC
GCGACGACATCGTTGTAAACCCTGATAATGTTGTAAGTAATGTGGGTGCTCATTTTGAAA

Male 5C (mutated: 5bp-deletion between black/blue positions in bold):
TATAGAGCGCAGTGTCAAT**CCAAGCTGT**TATTAAACGTAGATTTGGTAATTTTAAAAGCATATTTTTTTCTTT
GAAATTCATAAGTTATCAATTATCGATGGAAATGTATTCTATGGAGAACGTTTTACCCGATGAATGGGTGCAA
AAATTATTTTACCTTCAAATCTACAATCAACACACGCTAACTTTTGTGACTTGATCAACTCTCACCTGGAAAA
GCAACCAACTACAATCAACATTCTATGGGATAATCGACAAGAATAAAAATAAGGTCAGCAGCCATGACTTTTC
CATCATTTTGAATATACCTTATTTGTTTCGGGATTAATTGGGGGTCGGAAATCCTCTTGAATTCAGAAACGGG
AACCGGAGGAAGGTGCCGGTCTTTCAGAAAGCTGTGAAAAATACCAACATTTCTGCTGCCAAGAGCTCAATAA
GAAGTTTCAAAAATTGTCTTGGATGTTGCAGCTGTGGCTGCTAAGTAA

Truncated CcTRA isoform encoded by this Male 5C cDNA (42 aa) from an adult XX male showing also wild-type cDNA clones, hence being a mosaic:
MNMNITKASATTRKIRIEQNVPSGSVRKGPYAIERSVNPSCY

Male 5D
TATAGAGCGCAGTGTCAAT**CCA**AGCGAAGTTGTTATTAAACGTAGATTTGGTAATTTTAAAAGCATATTTTTT
TCTTTGAAATTCATAAGTTATCAATTATCGATGGAAATGTATTCTATGGAGAACGTTTTACCCGATGAATGGG
TGCAAAAATTATTTTACCTTCAAATCTACAATCAACACACGCTAACTTTTGTGACTTGATCAACTCTCACCTG
GAAAAGCAACCAACTACAATCAACATTCTATGGGATAATCGACAATGTGAAGGCTCAAAGCCTCTGTTTCAAC
GCGACGACATCGTTGTAAACCCTGATAATGTTGTAAGTAATGTGGGTGCTCATTTTGAAA

Male 6A
TATAGAGCGCAGTGTCAAT**CCA**AGCGAAGTTGTTATTAAACGTAGATTTGAACGTTTTACCCGATGAATGGGT
GCAAAAATTATTTTACCTTCAAATCTACAATCAACACACGCTAACTTTTGTGACTTGATCAACTCTCACCTGG
AAAAGCAACCAACTACAATCAACATTCTATGGGATAATCGACAATCTATGTAATAAGTAAACCAAGGAAAGGG

AAAATATCTACAATCAACAAGCCATTGTTGCAGCAACAAAGCAACTGAAACTACAATCAACATTCAATAAACT
TGGGTAATTTGGAATTTAATTCTCTGGGACACCTGTGGATTACAACAATCAACTCGAAACTTATTATACAATG
TGAAGGCTCAAAGCCTCTGTTTCAACGCGACGACATCGTTGTAAACCCTGATAATGTTGTAAGTAATGTGGGT
GCTCATTTTGAAA

Male 6B
TATAGAGCGCAGTGTCAAT**CCA**AGCGAAGTTGTTATTAAACGTAGATTTGGTAATTTTAAAAGCATATTTTTT
TCTTTGAAATTCATAAGTTATCAATTATCGATGGAAATGTATTCTATGGAGAACGTTTTACCCGATGAATGGG
TGCAAAAATTATTTTACCTTCAAATCTACAATCAACACACGCTAACTTTTGTGACTTGATCAACTCTCACCTG
GAAAAGCAACCAACTACAATCAACATTCTATGGGATAATCGACAATGTGAAGGCTCAAAGCCTCTGTTTCAAC
GCGACGACATCGTTGTAAACCCTGATAATGTTGTAAGTAATGTGGGTGCTCATTTTGAAA
