# Supplementary material for: Targeting the autosomal Ceratitis capitata transformer gene using Cas9 or dCas9 to masculinize XX individuals without inducing mutations
Source: BMC Genet. 2020 Dec 18;21(Suppl 2):150. doi: 10.1186/s12863-020-00941-4 (PMC7747381; doi:10.1186/s12863-020-00941-4)
Supplement: Supplementary file 3 — Additional file 3. [file 12863_2020_941_MOESM3_ESM.pdf]

Additional file n. 3

8 gDNA clone sequences from six XX females. The PAM sequence (antisense) is indicated in red bold.

Wild-type genomic locus *Cctra*
TATAGAGCGCAGTGTCAAT**CCA**AGCGAAGTTGTTATTAAACGTAGATTTGGTAATTTTAAAAGCATATTTTTT
TCTTTGAAATTCATAAGTTATCAATTATCGATGGAAATGTATTCTATGGAGAACGTTTTACCCGATGAATGGG
TGCAAAAATTATTTTACCTTCAAATCTACAATCAACACACGCTAACTTTTGTGACTTGATCAACTCTCACCTG
G

Female 1A
TATAGAGCGCAGTGTCAAT**CCA**AGCGAAGTTGTTATTAAACGTAGATTTGGTAATTTTAAAAGCATATTTTTT
TCTTTGAAATTCATAAGTTATCAATTATCGATGGAAATGTATTCTATGGAGAACGTTTTACCCGATGAATGGG
TGCAAAAATTATTTTACCTTCAAATCTACAATCAACACACGCTAACTTTTGTGACTTGATCAACTCTCACCTG
G

Female 1C
TATAGAGCGCAGTGTCAAT**CCA**AGCGAAGTTGTTATTAAACGTAGATTTGGTAATTTTAAAAGCATATTTTTT
TCTTTGAAATTCATAAGTTATCAATTATCGATGGAAATGTATTCTATGGAGAACGTTTTACCCGATGAATGGG
TGCAAAAATTATTTTACCTTCAAATCTACAATCAACACACGCTAACTTTTGTGACTTGATCAACTCTCACCTG
G

Female 2E
TATAGAGCGCAGTGTCAAT**CCA**AGCGAAGTTGTTATTAAACGTAGATTTGGTAATTTTAAAAGCATATTTTTT
TCTTTGAAATTCATAAGTTATCAATTATCGATGGAAATGTATTCTATGGAGAACGTTTTACCCGATGAATGGG
TGCAAAAATTATTTTACCTTCAAATCTACAATCAACACACGCTAACTTTTGTGACTTGATCAACTCTCACCTG
G

Female 3A
TATAGAGCGCAGTGTCAAT**CCA**AGCGAAGTTGTTATTAAACGTAGATTTGGTAATTTTAAAAGCATATTTTTT
TCTTTGAAATTCATAAGTTATCAATTATCGATGGAAATGTATTCTATGGAGAACGTTTTACCCGATGAATGGG
TGCAAAAATTATTTTACCTTCAAATCTACAATCAACACACGCTAACTTTTGTGACTTGATCAACTCTCACCTG
G

Female 4B
TATAGAGCGCAGTGTCAAT**CCA**AGCGAAGTTGTTATTAAACGTAGATTTGGTAATTTTAAAAGCATATTTTTT
TCTTTGAAATTCATAAGTTATCAATTATCGATGGAAATGTATTCTATGGAGAACGTTTTACCCGATGAATGGG
TGCAAAAATTATTTTACCTTCAAATCTACAATCAACACACGCTAACTTTTGTGACTTGATCAACTCTCACCTG
G

Female 4D
TATAGAGCGCAGTGTCAAT**CCA**AGCGAAGTTGTTATTAAACGTAGATTTGGTAATTTTAAAAGCATATTTTTT
TCTTTGAAATTCATAAGTTATCAATTATCGATGGAAATGTATTCTATGGAGAACGTTTTACCCGATGAATGGG
TGCAAAAATTATTTTACCTTCAAATCTACAATCAACACACGCTAACTTTTGTGACTTGATCAACTCTCACCTG
G

Female 5B

TATAGAGCGCAGTGTCAAT**CCA**AGCGAAGTTGTTATTAAACGTAGATTTGGTAATTTTAAAAGCATATTTTTT
TCTTTGAAATTCATAAGTTATCAATTATCGATGGAAATGTATTCTATGGAGAACGTTTTACCCGATGAATGGG
TGCAAAAATTATTTTACCTTCAAATCTACAATCAACACACGCTAACTTTTGTGACTTGATCAACTCTCACCTG
G

Female 6C
TATAGAGCGCAGTGTCAAT**CCA**AGCGAAGTTGTTATTAAACGTAGATTTGGTAATTTTAAAAGCATATTTTTT
TCTTTGAAATTCATAAGTTATCAATTATCGATGGAAATGTATTCTATGGAGAACGTTTTACCCGATGAATGGG
TGCAAAAATTATTTTACCTTCAAATCTACAATCAACACACGCTAACTTTTGTGACTTGATCAACTCTCACCTG
G
