# Supplementary material for: Targeting the autosomal Ceratitis capitata transformer gene using Cas9 or dCas9 to masculinize XX individuals without inducing mutations
Source: BMC Genet. 2020 Dec 18;21(Suppl 2):150. doi: 10.1186/s12863-020-00941-4 (PMC7747381; doi:10.1186/s12863-020-00941-4)
Supplement: Supplementary file 4 — Additional file 4. [file 12863_2020_941_MOESM4_ESM.pdf]

Additional file n. 4
Primer sequences

Primer Name	Sequence 5’--> 3’
Cctra 164+	CAGTGGTTCGGTTCGGAAG
Cctra 900-	TCCATGATGTCGATATTGTCC
Cctra 320-	TTTCAAAATGAGCACCCACA
CcSOD+	TGCTCCGAGAACGTTCACG
CcSOD-	TCATCGGTCAATTCGTGCAC
CcMoY A+	ACGGAACACATGCTAGCAGA
CcMoY A-	TTGCCCCAAAATTTCGGTTCC
T7-Cctra-164+	*TAATACGACTCACTATAGGGAGA*CAGTGGTTCGGTTCGGAAG
T7-Cctra-900-	*TAATACGACTCACTATAGGGAGA*TCCATGATGTCGATATTGTCC

F-sgtraEx1
GAAATTAATACGACTCACTATAGCGTTTAATAACAACTTCGCTgttttagagctagaaatagc

Reverse-Crispr
AAAAGCACCGACTCGGTGCCACTTTTTCAAGTTGATAACGGACTAGCCTTATTTTAACTTGCTATTTCTAGCTCTAAAAC
