# Supplementary material for: Targeting the autosomal Ceratitis capitata transformer gene using Cas9 or dCas9 to masculinize XX individuals without inducing mutations
Source: BMC Genet. 2020 Dec 18;21(Suppl 2):150. doi: 10.1186/s12863-020-00941-4 (PMC7747381; doi:10.1186/s12863-020-00941-4)

Additional file n.5
Raw unedited gels used in the Figures

Fig. 3A

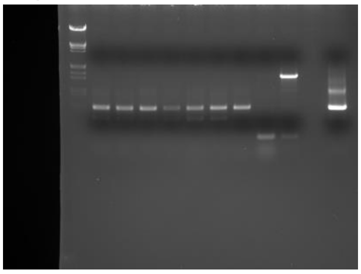

Fig. 3B

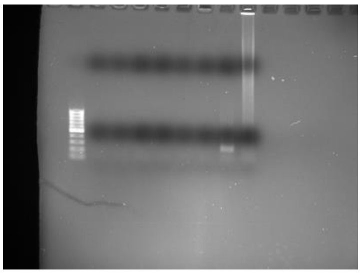

Fig. 4A-panel 1

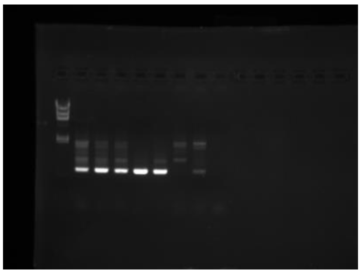

Fig. 4A-panel 2

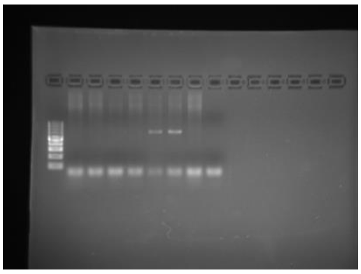

Fig. 4A-panel 3

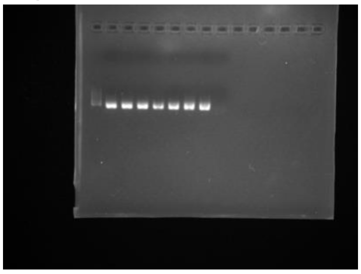

Fig. 4B- panel 1

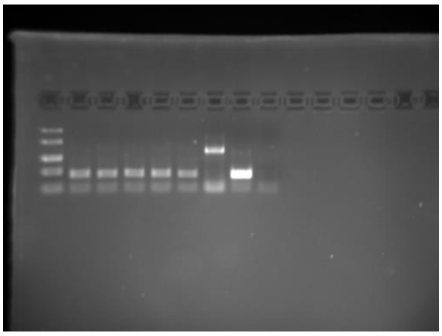

Fig. 4B- panel 2

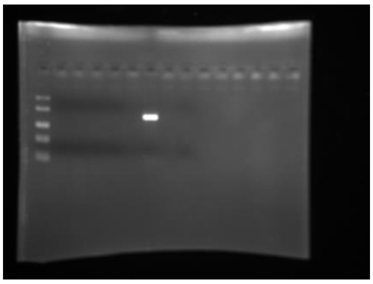

Fig. 4B- panel 3

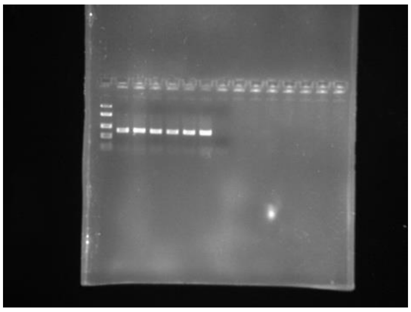

Fig. S2

Male1

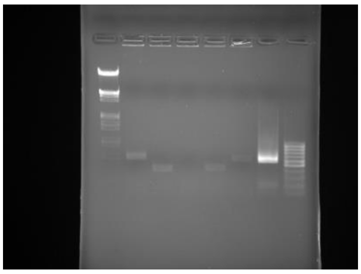

Male2

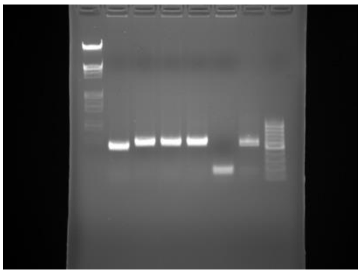

Male 3

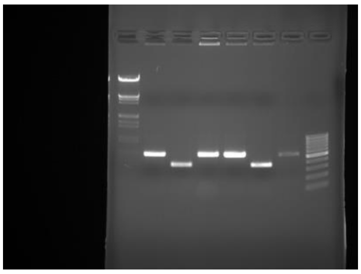

Male 4

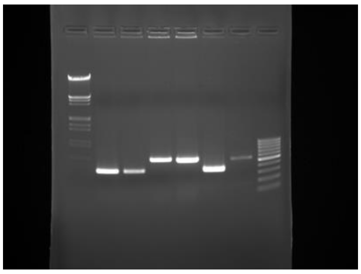

Male 5

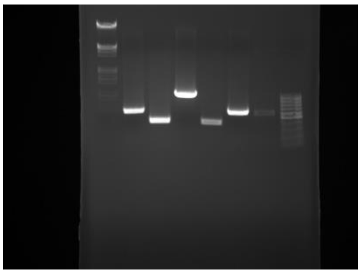

Male 6

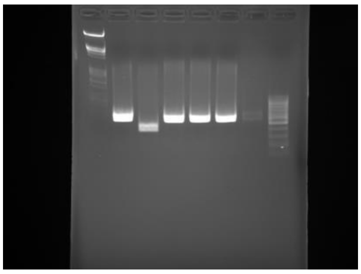

Fig. S4

Female 1 and 2

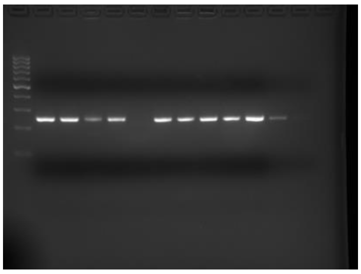

Female 3 and 4

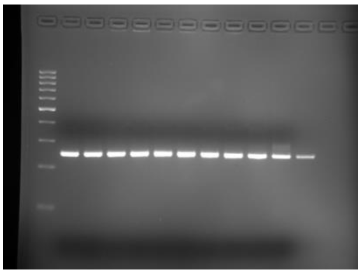

Female 5 and 6

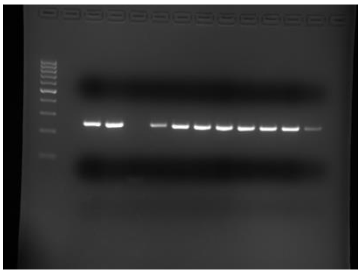

Fig. S5 B

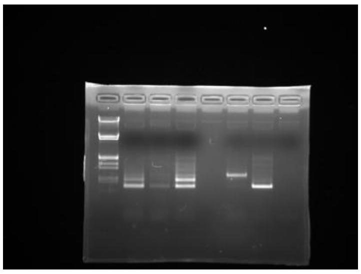

Supplement: Supplementary file 5 — Additional file 5. [file 12863_2020_941_MOESM5_ESM.pdf]
